# *Enterococcus faecalis* Bacteriophage vB_EfaS_efap05-1 Targets the Surface Polysaccharide and ComEA Protein as the Receptors

**DOI:** 10.3389/fmicb.2022.866382

**Published:** 2022-03-31

**Authors:** Lingqiong Huang, Wenqiong Guo, Jiahui Lu, Wuliang Pan, Fuqiang Song, Peng Wang

**Affiliations:** ^1^Yunnan Provincial Key Laboratory for Zoonosis Control and Prevention, Yunnan Institute of Endemic Diseases Control and Prevention, Dali, China; ^2^School of Public Health, Dali University, Dali, China; ^3^School of Nursing, Chengdu Medical College, Chengdu, China; ^4^School of Clinical Medicine, Chengdu Medical College, Chengdu, China; ^5^School of Pharmacy, Chengdu Medical College, Chengdu, China; ^6^Department of Medical Laboratory, The General Hospital of Western Theater Command, Chengdu, China

**Keywords:** bacteriophage, phage receptor, *Enterococcus faecalis*, exopolysaccharide, ComEA protein

## Abstract

*Enterococcus faecalis* is a Gram-positive opportunistic pathogen that causes nosocomial infections in humans. Due to the growing threat of antibiotic resistance of *E. faecalis*, bacteriophage therapy is a promising option for treating of *E. faecalis* infection. Here, we characterized a lytic *E. faecalis* bacteriophage vB_EfaS_efap05-1 with a dsDNA genome of 56,563 bp. Phage vB_EfaS_efap05-1 had a prolate head and a tail, and belongs to Saphexavirus which is a member of *Siphoviridae*. Efap05-1 uses either surface polysaccharide or membrane protein ComEA as the receptor because the mutation of both genes (*ComEA* and UDP-glucose 4-epimerase *galE*) prevents phage adsorption and leads to phage resistance, and complementation of *ComEA* or *galE* could recover its phage sensitivity. Our results provided a comprehensive analysis of a new *E. faecalis* phage and suggest efap05-1 as a potential antimicrobial agent.

## Introduction

*Enterococcus faecalis* is a gram-positive bacterium that could cause intestinal dysbiosis or infections in humans, such as nosocomial sepsis, urinary tract, and surgical site infections (Hayakawa et al., 2013; Jahansepas et al., 2018). In addition, the cytolysin-positive *E. faecalis* strains are correlated with mortality in patients with alcoholic hepatitis (Duan et al., 2019). However, the emergence of antibiotic resistance, especially vancomycin and daptomycin resistance, is especially troubling (Hu et al., 2018). Thus, new therapeutic approaches are needed to treat *E. faecalis* infections.

Bacteriophages are viruses that infect bacteria and are promising agents for antimicrobial treatment (Kortright et al., 2019; Wu et al., 2021). Recently, phage therapy clinical trials are initiated in many countries, and the number of case reports describing patients being treated increased significantly word-wide. For example, COVID-19 patients with carbapenem-resistant *Acinetobacter baumannii* infection were treated with a pre-optimized 2-phage cocktail and the infection was significantly relieved (Wu et al., 2021). However, phage resistance is a potential barrier to successful phage therapy (Egido et al., 2021). To confront this issue, the molecular mechanisms of phage resistance, as well as the genomic and biological characteristics of a phage, should be studied to provide the foundation for rational selection of the phages for therapy (Pires et al., 2020).

In this study, we isolated a new *E. faecalis* bacteriophage vB_EfaS_efap05-1 with a dsDNA genome of 56,563 bp. It belongs to Saphexavirus which is a member of *Siphoviridae*, and efap05-1 could use either surface polysaccharide or membrane protein ComEA as the receptor to adsorb to the bacterial surface. Thus, phage-resistant mutants had both mutations of *ComEA* and *galE*. In summary, this study provided a detailed characterization of an *E. faecalis* bacteriophage and suggests efap05-1 as a potential antimicrobial agent.

## Experimental Procedures

### Bacterial Strains and Phages

*Enterococcus* strains were collected from the Department of Clinical Laboratory Medicine and were grown aerobically on Brain-Heart Infusion Broth (BHI) broth at 37°C with shaking.

Bacteriophage was isolated from hospital sewage as previously described (Yang et al., 2016). Briefly, the sewage was pelleted, and the supernatant was filtered through a 0.22 μm-pore-size filter. Then, 500 μl of the sample was immediately mixed with 200 μl of bacterial culture, and 4 ml of molten BHI soft agar (0.4%) was added and poured onto BHI agar plates. After overnight culture, the formed plaque was picked, deposited in 1 ml of BHI, followed by a 10-fold dilution and double-layer agar assay to purify the phage. Then, one plaque from the third round of the purification process was picked for this study.

### Transmission Electron Microscopy

Phage particles were dropped on carbon-coated copper grids for 10 min. Then, the grids were stained with phosphotungstic acid (pH 7.0) for 15 s. The sample was examined under a Philips EM 300 electron microscope. The sizes of the phage were measured using AxioVision LE based on five randomly selected images.

### Phage Titering and MOI Experiment

The double-layer agar plate assay was used to calculate the phage titer. Briefly, 10-fold dilutions of phage solution were mixed with 200 μl of host bacteria, then mixed with 4 ml of molten BHI broth with 0.4% agar. Then, pour the mixture on a 1.5% agar plate. After overnight incubation at 37°C, the number of plaques was calculated as a plaque-forming unit (pfu). MOI experiments were performed by mixing log-phase bacteria (OD600 = 0.6) with a different number of phages, and the titer in the coculture was calculated using a double-layer agar plate assay after 5 h.

### One-Step Growth

The one-step growth curve of efap05-1 was determined as described (Zhong et al., 2020). Briefly, 1 ml of log-phase bacteria and 1 ml of efap05-1 were mixed at an MOI of 10 and incubated at 37°C for 10 min. Then, the mixture was centrifuged for 1 min at a speed of 10,000 × *g*, and the pellet was resuspended in 6 ml of BHI medium. And samples were taken at the given time points, which are immediately pelleted and phage titer in the supernatant was measured immediately.

### Adsorption Rate Experiments

Bacteriophage adsorption assay with various time points was performed as previously described (Al-Zubidi et al., 2019). Briefly, the log phase bacterial cultures were pelleted and resuspended in medium to a final concentration of 3 × 10^8^ CFU/ml. Then, phage was added to a final titer of 3 × 10^6^ pfu/ml. The samples were cultured at 37°C for 10 min, and the phage titer in the supernatant were measured using the double-agar plating assays. The adsorption rate was calculated as (the original phage titer—the remaining phage titer)/the original phage titer.

### Determination of Host Range

Ten *E. faecalis* strains were selected to test the host range of efap05-1 through spot testing by dropping 1 μl of phage onto the double-layer soft agar premixed with the bacterial and cultured at 37°C for overnight. The formation of a clear plaque is considered as sensitive to phage efap05-1 infection.

### EOP Assay

Two microliter of serial 10-fold dilutions of phage efap05-1 were spotted on double layer agar plates containing a bacterial host. The number of plaques observed after overnight incubation were compared to the number obtained on the strain efa05.

### Genome Sequencing and Annotation

The phage DNA extraction is performed as previously described (Khan et al., 2021). Then, phage genomic DNA was sequenced using an Illumina Hiseq 2,500 platform (~1 Gbp/sample). Fastp (Chen et al., 2018) was used for adapter trimming and filtering the raw reads. The data were assembled using the *de novo* assembly algorithm Newbler Version2.9 with default parameters, and the assembled genome was annotated using RAST (Overbeek et al., 2014). The DNA and protein sequences were checked for homologs with BLAST manually. The genome map was drawn by SnapGene 4.1.8. The sequence data is available in the NCBI under accession number OL505085.

### Stability Studies

The stability of phage under various conditions was tested by treating 10^9^ pfu of phage under different pH (pH 2–13), temperature (0°C, 30°C, 40°C, 50°C, and 60°C), or chloroform concentration (10%, 25%, 50%, 75%, and 95%) for 60 min, the then the titer of the phage was determined by double-layer agar assay.

### Selection of the Phage-Resistant Mutants

The phage resistant mutants were selected as previously described (Shen et al., 2018). The log phase bacteria were mixed with phage efap05-1 and cultured until the bacteria was lysed. Then, the lysate was inoculated onto the BHI agar. After overnight incubation, the single colonies were checked for its resistance against phage using the double-layer agar assay, which confirmed that all the colonies on the plates are resistant to phage infection.

### Bacterial Genome Sequencing

The wide type strain efa05 and phage resistant mutant strain efa05R were selected for sequencing. Bacterial genomic DNA was extracted using UNlQ-10 Column Bacterial Genomic DNA Isolation Kit (sangon bitotec: SK1202), and then sent to Novogene Corporation for sequencing using the Illumina Hiseq 2,500 platform. Trimmomatic was used to remove adapter sequences and low-quality bases (Bolger et al., 2014). BWA was used to map clean reads to the reference genome sequence of efa05. Samtools (Li et al., 2009) was then used to prepare the data for use with the Integrative Genomics Viewer (IGV). DNA mutation locations were manually checked with IGV and SeqKit (Shen et al., 2016).

### Complementation of *ComEA* and *galE*

The *ComEA* gene and plasmid pMGP23:mCherry were amplified by PCR (the primers for the amplification of *ComEA* gene and the plasmid pMGP23:mCherry are listed in Table 1), and the PCR products were purified. The *ComEA* and plasmid were ligated by Gibson assembly to generate pMG-ComEA, and the constructed plasmid was comfired by sanger sequencing. The efa05R complementation strain was generated by electroporation of pMG-ComEA into strain efa05R followed by selection on BHI agar erythromycin (20ug/ml). The *galE* gene was complemented with the same protocol.

**Table 1:** Bacterial strains, phages, plasmids, and primers used in this study.

Names	Characteristics and descriptions	Source
***Enterococcus faecalis***		
efa01	Human blood isolate	
efa02	Human blood isolate	
efa03	Human blood isolate	
efa04	Human blood isolate	
efa05	Human urine isolate	This study
efa06	Human urine isolate	
efa07	Human urine isolate	
efa08	Human urine isolate	
efa09	Human urine isolate	
efa10	Human urine isolate	
efa05R	phage resistant mutant	
efa05R::*ComEA*	Complementation of *ComEA* strain	
efa05R::*galE*	Complementation of *galE* strain	
**Phage**		
efap05-1	*Siphoviridae*; isolated from sewage	This study
**Plasmids**		
pMGP23	Modified from plasmid pMG36e, which contains erythromycin and kanamycin resistance gene	This study
pMG-ComEA	pMGP23 expressing *ComEA* from the native promoter	This study
pMG-galE	pMGP24 expressing *galE* from the native promoter	This study
**Primers**		
ComEA-F	aaaatattcggaggaattttgaaatggattggttgaaacagttac	
ComEA-R	atatcgtagcgccggttaacggtaaattctaatagcattctctttaca	
galE-F	atatcgtagcgccggtcatctctgcttaccttccg	
galE-R	aaaatattcggaggaattttgaagtggaatcatttctaatcacagg	
pMGP23-F	ccggcgctacgatatt	
pMGP23-R	ttcaaaattcctccgaat	

### Statistical Analysis

All the experiments were performed three times. The statistical analysis was performed using One-way ANOVA or *t*-test, and statistical significance was assumed if the value of *p* was <0.05.

## Results

### The Biological Characterization of an *Enterococcus faecalis* Phage

**Figure 1:**
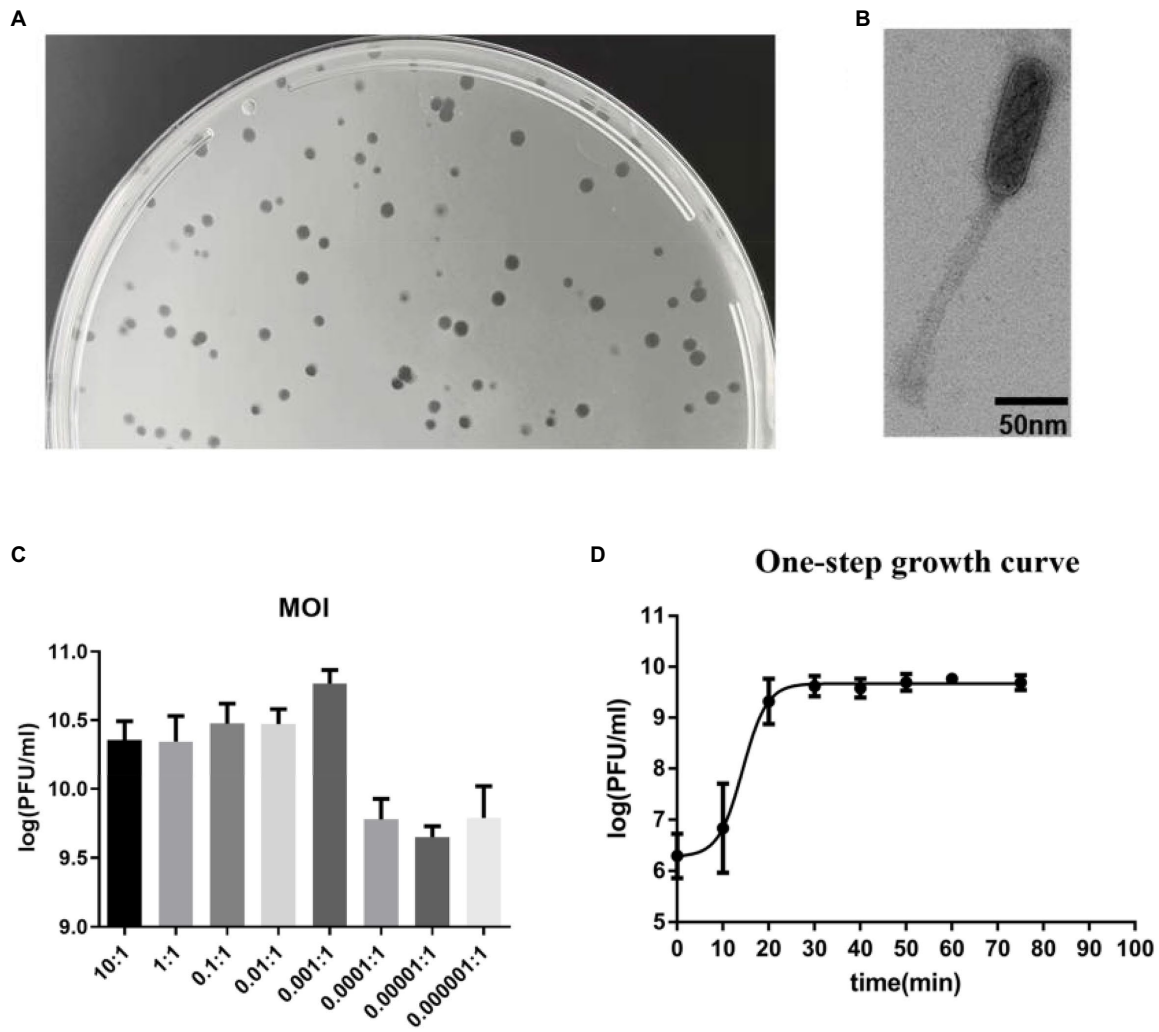
Biological characterization of *E. faecalis* phage vB_EfaS_efap05-1. **(A)** Phage efap05-1 forms clear plaques on the agar plate. **(B)** The transmission electron micrograph reveals that efap05-1 is a Saphexavirus which is a member of Siphoviridae. **(C)** The optimal MOI of phage efap05-1 is 0.001. **(D)** The one-step growth curve of efap05-1.

An *E. faecalis* phage was isolated using plaque assay. It forms a clear plaque on the host strain efa05 in the double layer agar plates (Figure 1A). The phage particle was observed by transmission electron microscopy. It is a non-enveloped, head-tail structural particle. The prolate head is approximately 100 nm in length and 40 nm in width (Figure 1B). Thus, based on the morphology, phage vB_EfaS_efap05-1 belongs to Saphexavirus which is a member of *Siphoviridae*. The optimal multiplicity of infection (MOI) was 0.001, and the phage titer could reach approximately 8*10^10^ pfu/ml (Figure 1C). The one-step growth curve of efap05-1 indicates that this phage replicates quickly with a lysis period of about 20 min, and the phage titer approached plateau after 20 min (Figure 1D), and the burst size was estimated as about 20 pfu per bacterium.

The host range of efap05-1 was estimated by EOP (efficiency of plating) assays. Ten clinically isolated *E. faecalis* strains were tested and five strains could be lysed by efap05-1, indicating a modest host range (Table 2).

**Table 2:** The host range of phage efap05-1.

Strain	Origin	LG1 sensitivity
*Enterococcus faecalis* efa01	Blood	−
*Enterococcus faecalis* efa02	Blood	−
*Enterococcus faecalis* efa03	Blood	+
*Enterococcus faecalis* efa04	Blood	−
*Enterococcus faecalis* efa05	Urine	+
*Enterococcus faecalis* efa06	Urine	+
*Enterococcus faecalis* efa07	Urine	−
*Enterococcus faecalis* efa08	Urine	−
*Enterococcus faecalis* efa09	Urine	+
*Enterococcus faecalis* efa10	Urine	+

+ Indicates the strain is sensitive to phage efap05-1, and EOP ranges between 0.001 and 1.

### Stability of efap05-1

**Figure 2:**
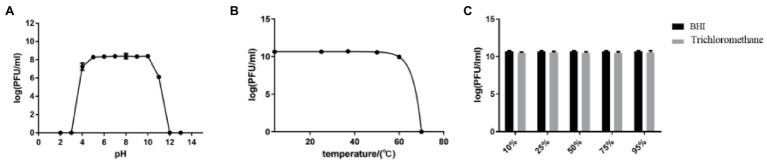
Stability of efap05-1: **(A)** Phage efap05-1 is stable under pH5 ~ 10, and is completely inactived under pH3. **(B)** Phage efap05-1 is inactivated by 70°C treatment. **(C)** Phage efap05-1 is resistant to chloroform because the titer was stable after chloroform treatment.

The stability of efap05-1 under various conditions was tested. It could maintain stability under pH 5–10, and other pH solutions could impair the viability of efap05-1 (Figure 2A). And efap05-1 is stable under 50°C because its titer was not changed after 60 min incubation at 50°C (Figure 2B), and is completely inactivated over 70°C. Besides, chloroform treatment did not affect the viability of phage efap05-1, indicating that it is a non-enveloped phage (Figure 2C).

### Genome Sequence Analysis of an *Enterococcus faecalis* Phage

**Figure 3:**
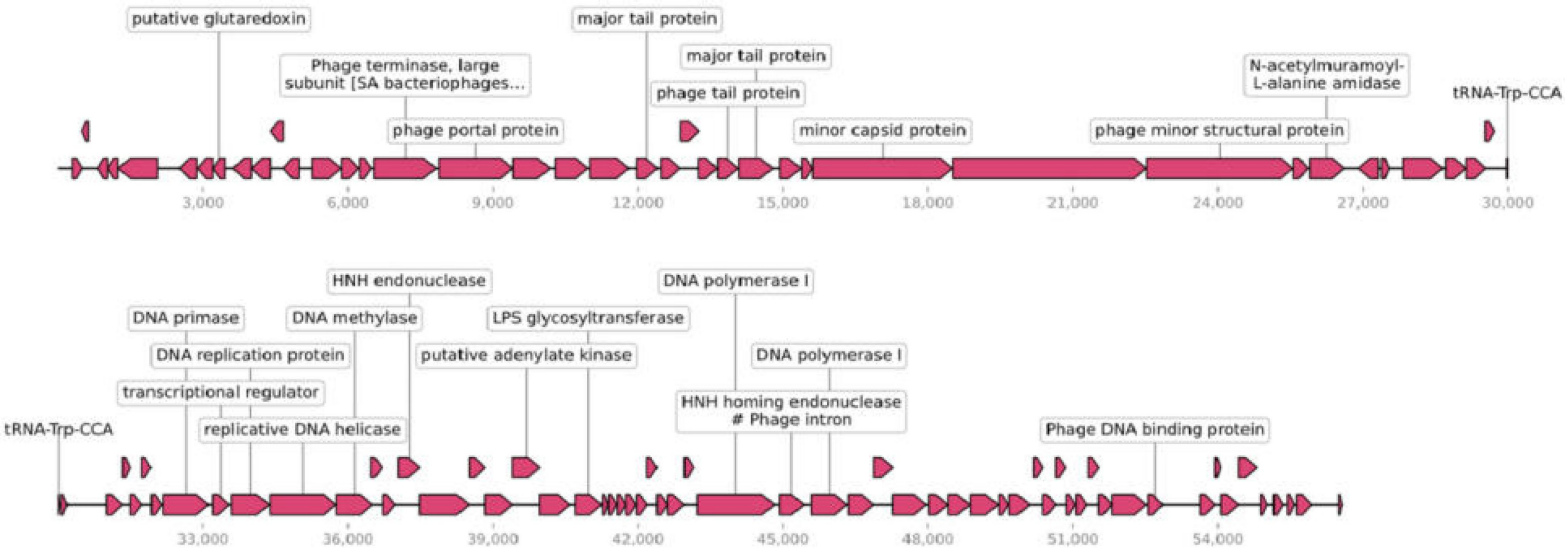
Genomic characterization of efap05-1. Phage efap05-1 is a dsDNA phage that encodes 99 predicted proteins and one tRNA.

Phage efap05-1 is a double-stranded (ds) DNA phage with a linear genome of 56,564 base pairs (bp). Its G + C content is 40% and encodes 99 ORFs and one tRNA (Figure 3), which are predicted by RAST (Overbeek et al., 2014) and visualized by SnapGene.

Most of the ORFs are functionally unknown, and 22 ORFs are functionally annotated, which can be categorized into several functional modules, including phage DNA replications, lysis, phage structural protein (Figure 3). However, efap05-1 did not encode any antibiotic-resistant gene or virulence gene, indicating that it is a safe candidate for phage therapy.

### Phage Resistant Mutant Contains Two Mutations

**Figure 4:**
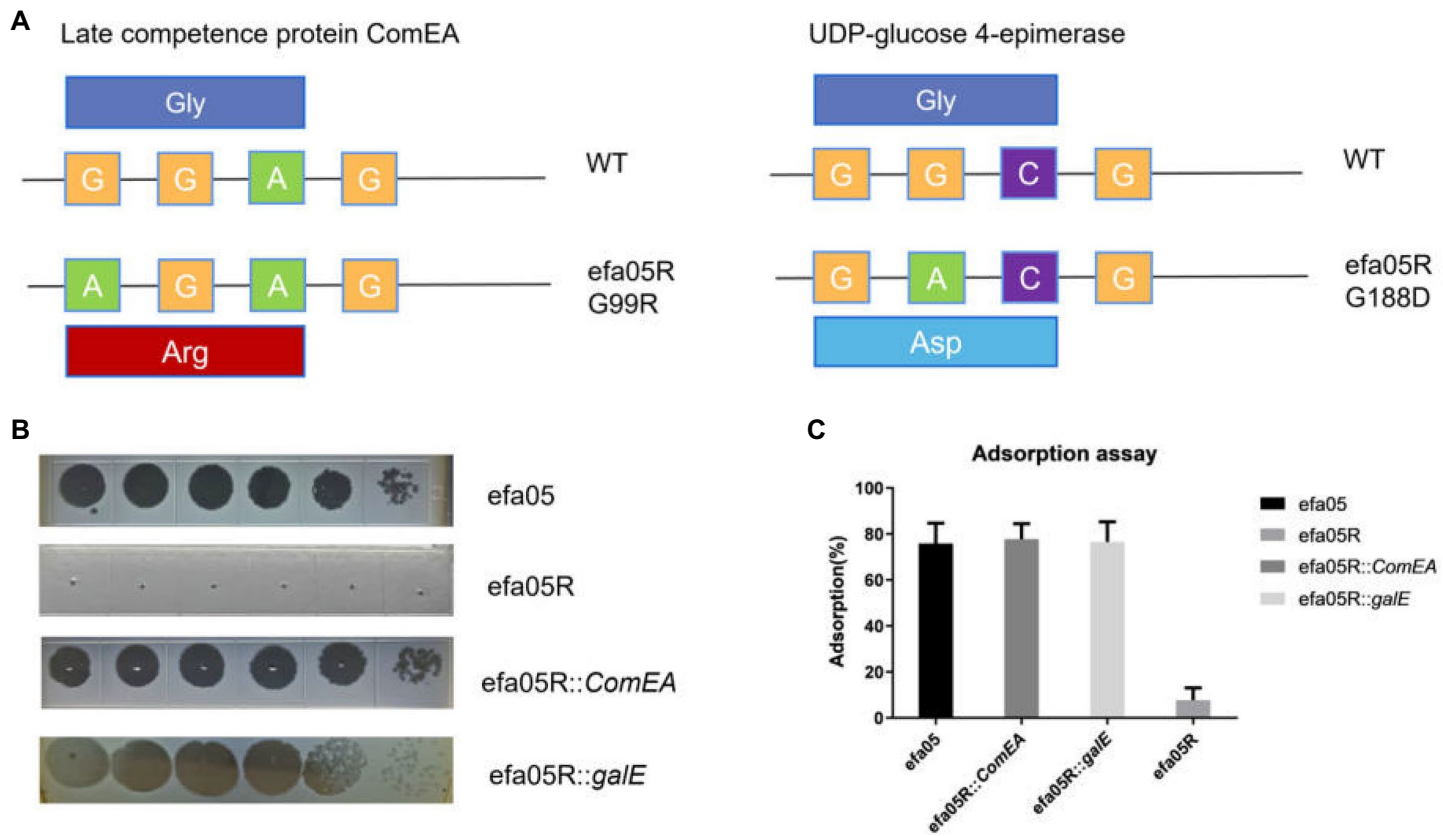
Characterization of the phage-resistant mutant. **(A)** The mutation site in efa05R was detected as G99R in *ComEA* and G188D in *galE*. **(B)** The EOP experiment of phage against wild type strain, phage resistant efa05R, and the complemented strains. **(C)** Adsorption assay of phage onto each strain.

To study the phage resistant mechanism of *E. faecalis* efa05 against phage efap05-1, we mixed phage with host and cultured until the bacteria are lysed. And then inoculate the lysate on BHI agar plate. One phage-resistant mutant efa05R was selected, and its resistance against efap05-1 was confirmed by EOP experiment (Figure 4B).

Then, efa05R and wild-type strain efa05 was sent for whole-genome sequencing, and the mutation sites of these two strains were detected as Late competence protein *ComEA* and UDP-glucose 4-epimerase *galE* (Figure 4A). ComEA is a cell membrane protein that binds to the double-stranded DNA and initiates the DNA uptake process (Burghard-Schrod et al., 2021). And UDP-glucose 4-epimerase is involved in the biosynthesis of cell wall polysaccharides (Boels et al., 2001; Lee et al., 2014).

Usually, phage resistance is selected with one key mutation site (Li et al., 2018), the mutation of both genes detected in phage resistant mutant efa05R indicates that both genes are required for phage infection. As expected, the complementation of either *ComEA* or *galE* in efa05R could recover the phage sensitivity through enabling phage adsorption (Figures 4B,C). Thus, these data indicate that phage could bind to either polysaccharides or protein ComEA to initiate the phage infection cycle.

## Discussion

*Enterococcus. faecalis* have developed resistance to antibiotics, including vancomycin and daptomycin (Hayakawa et al., 2013; Jahansepas et al., 2018). Thus, phage therapy is a renewed interest to treat multidrug-resistant *E. faecalis* infection. The biological and genomic characterization of a phage is essential before applications in phage therapy (Barbu et al., 2016; El Haddad et al., 2019; Pires et al., 2020). In this study, we isolated an *E. faecalis* bacteriophage vB_EfaS_efap05-1 with a prolate head. It is a completely lytic phage without the antibiotic-resistant genes or virulence genes, indicating it as a potential candidate for phage therapy.

The identification of phage resistance mechanisms is important for rational select phage or designing a phage cocktail for therapy (Labrie et al., 2010; Duerkop et al., 2016). Phage resistance is quite common and is important for phage therapy because it could lead to treatment failure. Selecting different phages that target different receptors is a rational approach in selecting phages for therapy (Yang et al., 2020). And *in vitro*, most phage resistance is selected through modifications of the receptors (Castillo et al., 2015; Duerkop et al., 2016). For example, *Pseudomonas aeruginosa* phage resistant mutants are O-antigen deficient to prevent phage adsorption (Shen et al., 2018). In this study, phage resistance mutants are selected with mutations in two genes. And the complementation of each gene could restore the phage sensitivity as well as the phage adsorption. Thus, it is reasonable to infer that phage efap05-1 uses either polysaccharides or protein ComEA as the receptors. Polysaccharides are common phage receptors for a lot of *E. faecalis* phages (Duerkop et al., 2016; Chatterjee et al., 2020).

ComEA is a membrane protein, and the loss of the ComEA decreases the binding of DNA to the competent cell surface and the internalization of DNA and impairs DNA transformability (Inamine and Dubnau, 1995). However, protein ComEA, to our knowledge, is the first report to serve as a phage receptor, which is an interesting biological phenomenon.

The limitation of this study is the lack of identification of the receptor binding proteins in the phages. Most phages use either polysaccharides or protein as receptors (Lim et al., 2021). However, it is not common that phage uses two different receptors of polysaccharides and membrane protein, because the phage tail fiber is very specifically targeting the receptors, and usually one phage tail fiber could not adsorb to two different structural receptors. And the *E.coli* phage phi92 could adsorb to both encapsulated and nonencapsulated bacteria due to the presence of four different types of tail fibers and tail spikes in the viral particles, which enable the phage to use attachment different sites on the host cell surface (Schwarzer et al., 2012). And staphylococcal Twort-like phage ΦSA012 possesses two receptor binding proteins to expand its host range (Takeuchi et al., 2016). In our study, the current data suggest that phage efap05-1 might also encode different receptor binding proteins that enable it to adsorb to both polysaccharides and membrane proteins. Three tail fiber proteins are annotated in the genome of efap05-1 (Figure 3), which needs further study to demonstrate the function of each tail fiber protein.

In conclusion, we isolated and characterized an *E. faecalis* phage efap05-1, which is a candidate for the development of phage cocktails or phage-antibiotic combinations treatment for *E. faecalis* infections. The characterization of the phage-resistant mutant bacterium could help to develop a cocktail to avoid phage resistance.

## Data Availability Statement

The datasets presented in this study can be found in online repositories. The names of the repository/repositories and accession number(s) can be found in the article/supplementary material.

## Author Contributions

PW and FS designed the research. LH, WG, JL, and WP performed the laboratory work and collected the data. LH, WG, PW, and FS wrote the first draft of the manuscript and prepared figures. All authors contributed to the article and approved the submitted version.

## Funding

This research was supported by the National Natural Science Foundation of China (NSFC, 31660043 to WP) and Yunnan Medical leading talent project (L-2019001 to WP).

## Conflict of Interest

The authors declare that the research was conducted in the absence of any commercial or financial relationships that could be construed as a potential conflict of interest.

## Publisher’s Note

All claims expressed in this article are solely those of the authors and do not necessarily represent those of their affiliated organizations, or those of the publisher, the editors and the reviewers. Any product that may be evaluated in this article, or claim that may be made by its manufacturer, is not guaranteed or endorsed by the publisher.

## References

[ref1] Al-Zubidi M. Widziolek M. Court E. K. Gains A. F. Smith R. E. Ansbro K.. (2019). Identification of novel bacteriophages with therapeutic potential that target *Enterococcus faecalis*. Infect. Immun. 87, e00512–e00519. doi: 10.1128/IAI.00512-19, PMID: 31451618 PMC6803325

[ref2] Barbu E. M. Cady K. C. Hubby B. (2016). Phage therapy in the era of synthetic biology. Cold Spring Harb. Perspect. Biol. 8:a023879. doi: 10.1101/cshperspect.a023879, PMID: 27481531 PMC5046696

[ref3] Boels I. C. Ramos A. Kleerebezem M. de Vos W. M. (2001). Functional analysis of the *Lactococcus lactis* galU and galE genes and their impact on sugar nucleotide and exopolysaccharide biosynthesis. Appl. Environ. Microbiol. 67, 3033–3040. doi: 10.1128/AEM.67.7.3033-3040.2001, PMID: 11425718 PMC92977

[ref4] Bolger A. M. Lohse M. Usadel B. (2014). Trimmomatic: a flexible trimmer for Illumina sequence data. Bioinformatics 30, 2114–2120. doi: 10.1093/bioinformatics/btu170, PMID: 24695404 PMC4103590

[ref5] Burghard-Schrod M. Kilb A. Kramer K. Graumann P. L. (2021). Single molecule dynamics of DNA receptor ComEA, membrane permease ComEC and taken up DNA in competent Bacillus subtilis cells. J. Bacteriol. doi: 10.1128/jb.00572-21, PMID: [Epub ahead of print]. 34928178 PMC8923214

[ref6] Castillo D. Christiansen R. H. Dalsgaard I. Madsen L. Middelboe M. (2015). Bacteriophage resistance mechanisms in the fish pathogen *Flavobacterium psychrophilum*: linking genomic mutations to changes in bacterial virulence factors. Appl. Environ. Microbiol. 81, 1157–1167. doi: 10.1128/AEM.03699-14, PMID: 25480749 PMC4292493

[ref7] Chatterjee A. Willett J. L. E. Nguyen U. T. Monogue B. Palmer K. L. Dunny G. M.. (2020). Parallel genomics uncover novel enterococcal-bacteriophage interactions. mBio 11, e03120–e031219. doi: 10.1128/mBio.03120-19, PMID: 32127456 PMC7064774

[ref606] Chen S. Zhou Y. Chen Y. Jia G. (2018). Fastp: an ultra-fast all-in-one fastq preprocessor. Bioinformatics 34, i884–i890. doi: 10.1093/bioinformatics/bty560, PMID: 30423086 PMC6129281

[ref8] Duan Y. Llorente C. Lang S. Brandl K. Chu H. Jiang L., 2019. . Bacteriophage targeting of gut bacterium attenuates alcoholic liver disease. Nature 575, 505–511, doi: 10.1038/s41586-019-1742-x, PMID: . 31723265 PMC6872939

[ref9] Duerkop B. A. Huo W. Bhardwaj P. Palmer K. L. Hooper L. V. (2016). Molecular basis for lytic bacteriophage resistance in Enterococci. mBio 7, e01304–e01316. doi: 10.1128/mBio.01304-16, PMID: 27578757 PMC4999554

[ref10] Egido J. E. Costa A. R. Aparicio-Maldonado C. Haas P. J. Brouns S. J. J. (2021). Mechanisms and clinical importance of bacteriophage resistance. FEMS Microbiol. Rev. 46:fuab048. doi: 10.1093/femsre/fuab048, PMID: 34558600 PMC8829019

[ref11] El Haddad L. Harb C. P. Gebara M. A. Stibich M. A. Chemaly R. F. (2019). A systematic and critical review of bacteriophage therapy against multidrug-resistant ESKAPE organisms in humans. Clin. Infect. Dis. 69, 167–178. doi: 10.1093/cid/ciy947, PMID: 30395179

[ref12] Hayakawa K. Marchaim D. Palla M. Gudur U. M. Pulluru H. Bathina P.. (2013). Epidemiology of vancomycin-resistant *Enterococcus faecalis*: a case-case-control study. Antimicrob. Agents Chemother. 57, 49–55. doi: 10.1128/AAC.01271-12, PMID: 23070173 PMC3535915

[ref13] Hu F. Zhu D. Wang F. Wang M. (2018). Current status and trends of antibacterial resistance in China. Clin. Infect. Dis. 67, S128–S134. doi: 10.1093/cid/ciy657, PMID: 30423045

[ref14] Inamine G. S. Dubnau D. (1995). ComEA, a Bacillus subtilis integral membrane protein required for genetic transformation, is needed for both DNA binding and transport. J. Bacteriol. 177, 3045–3051. doi: 10.1128/jb.177.11.3045-3051.1995, PMID: 7768800 PMC176991

[ref15] Jahansepas A. Ahangarzadeh Rezaee M. Hasani A. Sharifi Y. Rahnamaye Farzami M. Dolatyar A.. (2018). Molecular epidemiology of Vancomycin-resistant enterococcus faecalis and enterococcus faecium isolated from clinical specimens in the northwest of Iran. Microb. Drug Resist. 24, 1165–1173. doi: 10.1089/mdr.2017.0380, PMID: 29708837

[ref16] Khan F. M. Gondil V. S. Li C. Jiang M. Li J. Yu J.. (2021). A novel *Acinetobacter baumannii* bacteriophage Endolysin LysAB54 With high antibacterial activity Against multiple gram-negative microbes. Front. Cell. Infect. Microbiol. 11:637313. doi: 10.3389/fcimb.2021.637313, PMID: 33738267 PMC7960757

[ref17] Kortright K. E. Chan B. K. Koff J. L. Turner P. E. (2019). Phage therapy: a renewed approach to combat antibiotic-resistant bacteria. Cell Host Microbe 25, 219–232. doi: 10.1016/j.chom.2019.01.014, PMID: 30763536

[ref18] Labrie S. J. Samson J. E. Moineau S. (2010). Bacteriophage resistance mechanisms. Nat. Rev. Microbiol. 8, 317–327. doi: 10.1038/nrmicro2315 20348932

[ref19] Lee M. J. Gravelat F. N. Cerone R. P. Baptista S. D. Campoli P. V. Choe S. I.. (2014). Overlapping and distinct roles of *Aspergillus fumigatus* UDP-glucose 4-epimerases in galactose metabolism and the synthesis of galactose-containing cell wall polysaccharides. J. Biol. Chem. 289, 1243–1256. doi: 10.1074/jbc.M113.522516, PMID: 24257745 PMC3894311

[ref20] Li H. Handsaker B. Wysoker A. Fennell T. Ruan J. Homer N.. (2009). The sequence alignment/map format and SAMtools. Bioinformatics 25, 2078–2079. doi: 10.1093/bioinformatics/btp352 19505943 PMC2723002

[ref21] Li G. Shen M. Yang Y. Le S. Li M. Wang J.. (2018). Adaptation of Pseudomonas aeruginosa to phage PaP1 predation via O-antigen polymerase mutation. Front. Microbiol. 9:1170. doi: 10.3389/fmicb.2018.01170, PMID: 29910791 PMC5992289

[ref22] Lim A. N. W. Yen M. Seed K. D. Lazinski D. W. Camilli A. (2021). A tail fiber protein and a receptor-binding protein mediate ICP2 bacteriophage interactions with vibrio cholerae OmpU. J. Bacteriol. 203:e0014121. doi: 10.1128/JB.00141-21 33875544 PMC8316142

[ref23] Overbeek R. Olson R. Pusch G. D. Olsen G. J. Davis J. J. Disz T.. (2014). The SEED and the rapid annotation of microbial genomes using subsystems technology (RAST). Nucleic Acids Res. 42, D206–D214. doi: 10.1093/nar/gkt1226, PMID: 24293654 PMC3965101

[ref24] Pires D. P. Costa A. R. Pinto G. Meneses L. Azeredo J. (2020). Current challenges and future opportunities of phage therapy. FEMS Microbiol. Rev. 44, 684–700. doi: 10.1093/femsre/fuaa017, PMID: 32472938

[ref25] Schwarzer D. Buettner F. F. Browning C. Nazarov S. Rabsch W. Bethe A.. (2012). A multivalent adsorption apparatus explains the broad host range of phage phi92: a comprehensive genomic and structural analysis. J. Virol. 86, 10384–10398. doi: 10.1128/JVI.00801-12, PMID: 22787233 PMC3457257

[ref26] Shen W. Le S. Li Y. Hu F. Q. (2016). SeqKit: a cross-platform and ultrafast toolkit for FASTA/Q file manipulation. PLoS One 11:e0163962. doi: 10.1371/journal.pone.0163962 27706213 PMC5051824

[ref27] Shen M. Zhang H. Shen W. Zou Z. Lu S. Li G.. (2018). Pseudomonas aeruginosa MutL promotes large chromosomal deletions through non-homologous end joining to prevent bacteriophage predation. Nucleic Acids Res. 46, 4505–4514. doi: 10.1093/nar/gky160, PMID: 29514250 PMC5961081

[ref28] Takeuchi I. Osada K. Azam A. H. Asakawa H. Miyanaga K. Tanji Y. (2016). The presence of two receptor-binding proteins contributes to the wide host range of staphylococcal Twort-Like phages. Appl. Environ. Microbiol. 82, 5763–5774. doi: 10.1128/AEM.01385-16, PMID: 27422842 PMC5038044

[ref29] Wu N. Dai J. Guo M. Li J. Zhou X. Li F. (2021). Pre-optimized phage therapy on secondary *Acinetobacter baumannii* infection in four critical COVID-19 patients. Emerg. Microb. Infect. 10, 612–618. doi: 10.1080/22221751.2021.1902754, PMID: 33703996 PMC8032346

[ref30] Yang Y. H. Lu S. G. Shen W. Zhao X. Shen M. Y.. (2016). Characterization of the first double-stranded RNA bacteriophage infecting *Pseudomonas aeruginosa*. Sci. Rep. 6:38795. doi: 10.1038/srep38795, PMID: 27934909 PMC5146939

[ref31] Yang Y. Shen W. Zhong Q. Chen Q. He X. Baker J. L.. (2020). Development of a bacteriophage cocktail to constrain the emergence of phage-resistant *Pseudomonas aeruginosa*. Front. Microbiol. 11:327. doi: 10.3389/fmicb.2020.00327, PMID: 32194532 PMC7065532

[ref32] Zhong Q. Yang L. Li L. Shen W. Li Y. Xu H.. (2020). Transcriptomic analysis reveals the dependency of *Pseudomonas aeruginosa* genes for double-stranded RNA bacteriophage phiYY infection cycle. iScience 23:101437. doi: 10.1016/j.isci.2020.101437 32827855 PMC7452160

